# Measuring total hip arthroplasty liner wear using the EOS Imaging System: experimental and clinical results

**DOI:** 10.2340/17453674.2024.41912

**Published:** 2024-09-13

**Authors:** Kristian KJÆRGAARD, Sibel YILMAZ, Bart KAPTEIN, Søren OVERGAARD, Ming DING

**Affiliations:** 1 Orthopaedic Research Unit, Department of Orthopaedic Surgery and Traumatology, Odense University Hospital, Odense, Denmark; 2 Department of Clinical Research, University of Southern Denmark, Odense, Denmark; 3 Biomechanics and Imaging Group, Department of Orthopedics, Leiden University Medical Center, Leiden, The Netherlands; 4 Copenhagen University Hospital, Bispebjerg Department of Orthopaedic Surgery and Traumatology, Frederiksberg Hospital, Copenhagen, Denmark; 5 Department of Clinical Medicine, Faculty of Health and Medical Sciences, University of Copenhagen, Copenhagen, Denmark

## Abstract

**Background and purpose:**

The low-dose EOS Imaging System is an emerging tool for 3-dimensional measurements in orthopedics. The clinical feasibility for measuring total hip arthroplasty (THA) liner wear has not yet been investigated. We aimed to evaluate the feasibility of using EOS to measure THA liner wear by examining the experimental accuracy using a THA phantom and clinical precision of patients with THA, considering a clinically relevant precision at the 95% repeatability limit to be 0.2 mm.

**Methods:**

An experimental THA phantom with movable stem and a fixed cup with a plastic liner was constructed to simulate progressive 3D liner wear. Series of 11 pairs of radiographs with 50 μm femoral movement in between were obtained for each 3D axis in EOS. 30 patients with a THA were scanned twice using EOS to assess precision. Model-based radiostereometric analysis (RSA) was used for wear measurement.

**Results:**

The mean difference (true minus simulated wear) with standard deviation (SD) and 95% limits of agreement for experimental THA wear were 0.005 (0.037) and [–0.069 to 0.079] mm for the vertical (y) axis. The mean (SD) and 95% repeatability limit for precision for clinical measurement were –0.029 (0.105) and 0.218 mm.

**Conclusion:**

Experimental THA liner wear using EOS was within clinically relevant tolerances and without bias. The clinical precision was just outside our defined clinically relevant precision. Compared with conventional RSA, EOS is less accurate and precise but may still be of value for certain clinical applications, provided larger sample size or longer follow-up are available.

The continuous component wear and migration in total hip arthroplasty (THA) are of particular interest, as these have been linked with early revision due to aseptic loosening [1]. Wear can be measured using radiostereometric analysis (RSA) with an accuracy of 0.05 mm [2,3]. Earlier research has shown that liner wear beyond 0.2 mm per year leads to a higher revision rate [1]. However, some newer liners with a wear rate of 0.09 mm per 5 years have been shown a higher revision rate than liners with an even lower wear rate [4,5]. We consider a precision at the 95% repeatability limit lower than 0.2 mm to be clinically relevant, and this is consistent with the precision reported in current RSA wear studies [6,7].

The current gold standard in continuous wear measurement is RSA [8]. 2 radiographs are taken from different angles simultaneously, and spatial information can be extracted from the radiographs by identifying implanted tantalum markers or the geometrical shape of implants [9,10]. The conventional RSA method requires specialist equipment and training and is time-consuming, and alternative methods may be of interest if accuracy and precision are within relevant tolerances.

The EOS Imaging System (EOS, Roubaix, France) is a biplane scanner that is used mainly in scoliosis diagnostics [11]. It is based on 2 vertically moving X-ray sources that create 2 perpendicular pushbroom projections on corresponding moving horizontal line detectors. The possibility for simple and fast stereo-image acquisition with the patient in a weightbearing position makes it interesting to explore EOS as an alternative to conventional RSA for THA liner wear measurement. Currently, the EOS imaging system has been tested for model-based RSA for unicompartmental knee arthroplasties and marker-based total knee arthroplasties, but not THA liner wear [12,13]. The use of EOS for clinical RSA studies has not yet been adopted for THA by the scientific community, and this study may in part address why.

We aimed to evaluate the feasibility of using EOS over conventional RSA to measure THA liner wear by examining the experimental accuracy using a THA phantom to investigate the instrument’s bias, and to examine the clinical precision of patients with THA to investigate smallest detectable clinical change.

## Methods

This study was prepared in accordance with Standards for Reporting Diagnostic accuracy studies (STARD) [14]. This study was approved by the Regional Committee of Southern Denmark on Health Science Ethics (S-20190069) and conducted following the Declaration of Helsinki.

### Terminology

We used definitions for accuracy and precision according to the RSA guideline [8]. Accuracy is defined as the mean difference between the measured and the true value. Precision is synonymous with repeatability and is defined as the mean difference between 2 independent measurements of the same values. Liner wear is measured as vertical (y-axis) head penetration into the cup.

### Experimental methods

#### Phantom construction

A vertical plate of 1-inch-thick acrylic glass was glued onto a similar horizontal plate (Figure). Half of a pelvis (Sawbones; https://www.sawbones.com/) with a THA cup and a plastic liner was attached to the vertical plate. A manual XYZ stage (Daedal Hannifin 4546M; Parker Hannifin Corp, Cleveland, OH, USA) with 10 μm indicators was attached to the horizontal plate. A femur (Sawbones) with a stem and a femoral head was attached to the platform of the stage using a clamp. In this way, the head could move freely independently of the cup with a small air gap between the head and the liner. All parts of the phantom were firmly attached with no play between parts.

**Figure:**
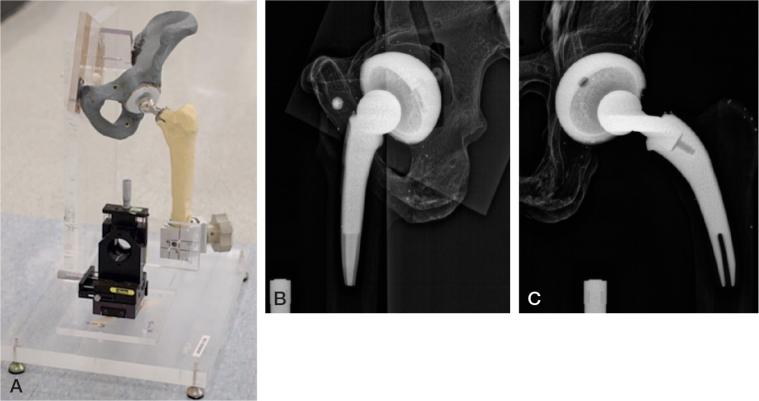
Hip phantom with a pelvis mounted on acrylic glass attached to a base plate of acrylic glass. A manual stage holding the femur is also mounted on the base plate. (B) Image recorded on the lateral (left) detector. (C) Image recorded on the frontal (right) detector.

#### Coordinate system

The axis configuration of the EOS setup was defined as follows: with a 3-dimensional right-hand coordinate system, the x-axis (anterior) was parallel to a horizontal line on the frontal (right) detector pointing away from the lateral detector, the y-axis (vertical) was parallel to a vertical line pointing upwards, and the z-axis (lateral) was parallel to a horizontal line on the lateral (left) detector pointing away from the frontal detector. The origin of the coordinate system was defined as the isocenter with the y-plane intersecting the floor of the machine.

#### Femoral movement and image acquisition

Image acquisition was done in 3 series of 11 image pairs, 1 series for each axis and 50 µm femoral movement between each pair. The phantom was placed in the EOS isocenter and the head was moved away from the cup/liner to allow free and independent head movement relative to the cup to simulate liner wear. To eliminate initial play, the stage platform was first moved 10 µm forward along each axis. At this position, the initial scan was performed. The stage platform was then moved 50 µm along the lateral axis and another scan was performed. This was repeated 10 times. After the last movement along the lateral axis, the same procedure was applied for the anterior and vertical axis. The phantom base plate was not moved during image acquisition, only the stage.

The EOS image acquisition parameters were as follows: both planes active, normal morphotype, scan speed at 4 (76 mm/s), exposure at 65 kV and 200 mA for both planes, and reference planes set to the isocenter (regardless of phantom position). Image resolution on the detector was 100 dots per inch (DPI).

#### Liner wear measurement using model-based RSA

Liner wear measurement was calculated using the EOS module (Izaak Walton Killam IWK Health Centre, Halifax, Nova Scotia, Canada) for model-based RSA (MBRSA, Model-based RSA 4.2, RSAcore, LUMC, Leiden, The Netherlands).

Liner wear measurement was calculated from an image pair to all subsequent image pairs (0–1, 0–2, …, 0–10, 1–2, …, 1–10, …, 9–10, n = 55 comparisons in total). The number of movements was based on previous research [15].

### Clinical methods

#### Participants

A cohort of patients with a THA, who were enrolled in a wear study and were visiting for their 10-year assessment, were included for test–retest measurements [4]. The visits took place from November 2019 to February 2020 at Odense University Hospital, Odense, Denmark. The inclusion and exclusion criteria for this study and the original wear study are stated in Table 1 (see Appendix).

**Table 1:** Inclusion and exclusion criteria

**Inclusion criteria**
The patient is considered eligible for THA by an orthopedic surgeon ^**a**^
Reason for eligibility is unilateral primary idiopathic osteoarthrosis ^**a**^
Choice of prosthesis is uncemented THA ^**a**^
Age 40–70 years at the time of inclusion in wear study ^**a**^
**Exclusion criteria**
Severe anteversion of femoral neck ^**a**^
Dysplasia with center–edge angle < 20° ^**a**^
Malignancy ^**a**^
Previous radiotherapy ^**a**^
Any kind of physical or psychological illness that renders it impossible for the patient to take part in our usual rehabilitation program ^**a**^
Complications during surgery that require the use of shell screws or cerclage around femur ^**a**^
Not being able to enter and exit EOS safely without walking aids
Not being able to stand still for 1 minute inside EOS.

a Original inclusion and exclusion criteria for the trial from which this study included patients.

The patients received a THA with an Exceed ABT cup (Biomet, Warsaw, IN, USA), ArComXL or E-Poly liner (Biomet), a Bi-Metric collarless titanium stem, and a CoCr femoral head (32 mm or 36 mm) according to the manufacturer’s instructions. Pain management, thromboprophylaxis, antibiotics, rehabilitation, and discharge were managed as usual.

12 females and 18 males were enrolled (Table 2). All had Exceed ABT cups, Bi-Metric stems, Co-Cr heads, and either an ArComXL cross-linked liner or an E-Poly cross-linked and oxidatively stabilized liner, all from Zimmer-Biomet. Median age was 71 when scanned and the distribution of left and right hip was equal.

**Table 2:** Descriptive data of 30 patients included for clinical tests

Age when scanned, median (IQR)	71 (65–75)
Sex: female/male	12/18
Side: right/left	15/15
Cup: Exceed ABT	30
Liner: Arcom XL/Epoly	15/15
Head: Co-Cr	30
Stem: Bi-Metric collarless	30
Cup size, mm	
52	2
54	3
56	10
58	4
60	9
62	1
64	0
66	1

#### Image acquisition and analysis

The patient was instructed to stand with their THA in the isocenter of EOS, facing out of the scanner with a 45° angle towards the frontal and lateral detectors, to stabilize their body by placing their hands on each wall, and to distribute the weight evenly across both legs with the THA leg rotated 15° internally. The anatomical area was set for pelvis and the scans were obtained witha 120 kV/200 mA setting. Scan speed was set to max (setting 1, 306 mm/s) to minimize the risk of movement during the scan. After the 1st scan, the patient was asked to step out of the scanner, and then step back inside to repeat the above for the 2nd scan.

Liner wear was calculated using MBRSA using the model-based approach for hip wear [16]. The difference was expressed as last minus first head–cup center distance for each axis. As no wear is expected to take place between scans, this approach can be used to assess clinical precision.

#### Sample size

The sample size calculation was based on marker-based RSA. This study was powered to achieve the same or lower standard error of the mean (SEM) as reported for conventional marker-based RSA: between 27.8 (3.1) and 54.8 (6.1) µm (mean of residuals [SD of that estimate] with n = 80 observation) [17]. The SD of the measurement error for marker-based RSA in EOS was between 5.2 um and 26.4 µm (further data from this study depends on axis and phantom location; data not shown). Knowing that SEM*√n = SD <–> n = (SD/SEM)^2^, we used SD = 26.4 (highest SD found for marker-based EOS in this study; data not shown) and SEM = 6.1 (highest SEM reported by the cited RSA precision study), and solved for n, which gives n = 18.7. The image quality of EOS scans for the purpose of this study was unknown, so the aim was to include 30 patients to ensure at least 20 useful scans.

### Statistics

For the experimental setup, the liner wear was calculated as applied translation minus measured translation. Rotation and translation in remaining axes were not considered. Results were reported as average error (accuracy, bias), SD of the error (precision, variance) and 95% limits of agreement (LoA, average error ± t × SD, where t = 2.0 is the 2-sided 95% t-statistic for n = 55).

For the clinical setup, descriptive data for the study population was given as median with quartiles or mean with 95% confidence intervals (CI) for skewed or normally distributed continuous data. Count data were reported as number and percentage.

Precision was calculated as 95% repeatability limit according to Ranstam et al. for an instrument without bias and is reported as average error, SD, and 95% and 99% repeatability limit of distance in mm using the relevant t-statistic (t = 2.0 for 95% confidence, t = 2.8 for 99% confidence, both at n = 30) [18]. Statistical analysis was performed using R version 4.4.0 (R Foundation for Statistical Computing, Vienna, Austria).

### Ethics, data sharing plan, funding, use of AI, and disclosures

This study was approved by the Regional Committee of Southern Denmark on Health Science Ethics (S-20190069) and conducted following the Declaration of Helsinki.

Data from the experimental part of this study will be made available upon reasonable request. Data from the clinical part of this study are not made available to protect patient confidentiality.

This study was supported by the Danish Orthopaedic Society, Danish Physicians’ Insurance of SEB Pension, Graduate School of Health Sciences at University of Southern Denmark, and Odense University Hospital, Denmark. The EOS module for MB-RSA was made available free of charge for this study. AI was not used in this study. The authors declare no further conflicts of interest. Complete disclosure of interest forms according to ICMJE are available on the article page, doi: 10.2340/17453674.2024.41912

## Results

### Experimental part

The accuracy was between –0.014 and 0.005 mm. The SD was between 0.037 and 0.076 with the lowest value for the vertical (y) axis, which is also the direction of most wear (Table 3). The 95% LoA were widest for the lateral axis (–0.149 to 0.156 mm) and narrowest for the vertical axis (–0.069 to 0.079 mm). The mean error was generally substantially lower than the SD.

**Table 3:** Experimental differences between measured and simulated THA liner wear for 55 measurements. Axes aligned to physical axes of EOS

Axis	Difference, mm mean (SD)	LoA, mm
X (anterior)	–0.014 (0.040)	[–0.094 to 0.066]
Y (vertical)	0.005 (0.037)	[–0.069 to 0.079]
Z (lateral)	0.003 (0.076)	[–0.149 to 0.156]

LoA = 95% limits of agreement.

### Clinical part

Precision as the 95% repeatability limit was 0.246 mm for the medial (x) axis, 0.218 mm for the vertical (y) axis, and 0.217 mm for the anterior (z) axis (Table 4). The most precise axis was again the anterior axis closely followed by the vertical axis.

**Table 4:** Precision (mm) as the 95% and 99% repeatability limit for repeated clinical THA liner wear measurements with 30 patients. Axes aligned to conventional RSA axes

Axis	Mean error (SD)	Precision	
		95%	99%
X (lateral)	0.017 (0.121)	0.246	0.332
Y (vertical)	–0.029 (0.105)	0.218	0.294
Z (anterior)	–0.006 (0.108)	0.217	0.292

## Discussion

We investigated the experimental accuracy and clinical precision of THA liner wear measurements using a THA phantom and patients with THA in repeated scans obtained using the EOS Imaging System. We showed that the experimental accuracy was within clinically relevant tolerances of 0.2 mm, but the clinical precision was just outside at 0.218 mm for the 95% repeatability limit. Compared with conventional RSA, EOS is less accurate and precise but may still be of value for clinical applications whenever EOS is available—simplicity of the measurement device is key—provided that a larger sample size with a longer follow-up is available.

The vertical axis is the most relevant axis for weight-bearing orthopedic implants, as subsidence, migration, and liner wear are most pronounced in this direction. Among the 3 axes, the narrowest experimental LoA was found for the vertical axis (–0.069 mm to 0.079 mm). Vertical axis motion is detectable on both the frontal and the lateral image as it corresponds with the vertical direction which is “in-plane” for both images. This would in theory increase the available data used for wear analysis, opposite to motion along the anterior or lateral axes, which is only detectable on either the frontal or lateral detector, respectively. For the clinical precision we chose to report only proximal wear and not 3-dimensional wear as lateral and anterior movement is measured only on 1 detector, hence we would expect a substantial influence from errors in the horizontal measurements. As such, we chose the simple and clinically most relevant measurement. However, given that the LoA for the anterior axis was somewhat similar (–0.094 to 0.066) and the lateral axis was larger (–0.149 to 0.156), this theory does not explain the data fully.

An earlier study has indicated that more than 0.2 mm liner wear per year may lead to a higher revision rate; however, this study, being the best current knowledge on osteolysis threshold, is from 2002 and may not reflect the performance of cross-linked polyethylene of today [1]. Based on results from our study, any liner wear would be detectable in the 2nd year after THA surgery, as it would be impossible to determine whether the measured difference was noise or actual movement occurring in the first year due to the precision of 0.218 mm. Howie et al. investigated proximal model-based RSA THA liner wear in an experimental setup similar to this and found an SD of 0.021, which is lower than our 0.037, so while no statistical comparison is directly available, the accuracy of conventional RSA is expected to be superior [19]. In recent clinical studies on liner wear, Nebergall et al. found a clinical precision of 0.16 mm for liner wear for the vertical axis [6], whereas Salemyr et al. reported a clinical precision of 0.19 mm at a 99% confidence interval [7] for their conventional RSA setups. This implies that a larger sample size or longer follow-up (e.g., more wear) is needed to achieve the same statistical certainty compared with conventional RSA in the assessment of THA liner wear. Given that dislocation, fracture, and prosthetic joint infection are now common causes for revision, it may be time for a renewed review on the osteolysis threshold [9].

### Strengths and limitations

The most notable limitations are the use of an uncalibrated manual stage, the use of theoretical image calibration, low detector resolution, and potential motion-induced blur in clinical images.

This study is limited by the use of theoretical image calibration based on values in the DICOM headers, and an optical calibration method for pushbroom projection has been made available since completion of the experiments in this study [20]. Optical image calibration may distribute the errors more evenly and provide a narrower LoA for some motion types, motion axes, or phantom positions. However, the accuracy was generally comparable to that of conventional RSA [15,21].

The EOS detector pixel width is 0.254 mm (100 DPI), and each motion step was 0.050 mm—or about one 5th of the pixel width. This resolution is lower than the recommended 150 DPI by MBRSA.

Image quality is directly related to liner wear measurement accuracy. The scan speed for the clinical precision measurements was set to the highest speed to minimize any movement during scanning. At this speed, it would take 0.7 seconds for the scanner to pass the cup and head. Given a desired precision of 0.2 mm we speculated that it was not unlikely that patient movement would reduce the precision, and we chose the highest scan speed. However, some images presented with a horizontally striped artifact, which challenged the contour detection algorithm and reduced the available data for the contour fit. These artifacts were not visible on the EOS monitor and were first noticed at the time of analysis after all scans were completed. It was unclear whether this was a result of the high scan speed or mechanical limitations. Furthermore, the high scan speed could potentially result in a higher standard deviation following lower image quality. In retrospect, the clinical scan speed should have been subject to a pilot test, but as our ethics approval did not cover initial trial and error this was not possible in the current study.

Future research could investigate the impact of correcting patient movement to improve image quality.

### Conclusion

Experimental THA liner wear using EOS was within clinically relevant tolerances and without bias. The clinical precision was just outside our defined clinically relevant precision. Compared with conventional RSA, EOS is less accurate and precise but may still be of value for certain clinical applications, provided larger sample size or longer follow-up are available.

## Notes

The study was conceptualized by KK, SO, and MD, experiments were performed by KK, SY, data were analyzed by KK, SY, BK, and MD. The manuscript was drafted by KK and SY, and all authors took part in the critical revision and approved of the final version.

The authors would like to acknowledge the time and effort provided by Charles Bragdon from Harris Orthopedics Laboratory at Massachusetts General Hospital in Boston, USA, where the experimental part of this study was performed.

Handling co-editors: Bart A. Swierstra and Jonas Ranstam

*Acta* thanks Dennis Janssen and Stephan Maximilian Röhrl for help with peer review of this manuscript.
